# Age structuring and spatial heterogeneity in prion protein gene (*PRNP*) polymorphism in white-tailed deer

**DOI:** 10.1080/19336896.2020.1832947

**Published:** 2020-10-20

**Authors:** Tyler K. Chafin, Marlis R. Douglas, Bradley T. Martin, Zachery D. Zbinden, Christopher R. Middaugh, Jennifer R. Ballard, M. Cory Gray, Don White, Michael E. Douglas

**Affiliations:** a Department of Biological Sciences, University of Arkansas, Fayetteville, AR, USA; b Arkansas Game and Fish Commission, Research, Evaluation, and Compliance Division, Little Rock, AR, USA; c University of Arkansas Agricultural Experiment Station, Monticello, AR, USA

**Keywords:** Adaptive management, chronic-wasting disease, demography, disease susceptibility, haplotype, selection, white-tailed deer

## Abstract

Chronic-wasting disease (CWD) is a prion-derived fatal neurodegenerative disease that has affected wild cervid populations on a global scale. Susceptibility has been linked unambiguously to several amino acid variants within the prion protein gene (*PRNP*). Quantifying their distribution across landscapes can provide critical information for agencies attempting to adaptively manage CWD. Here we attempt to further define management implications of *PRNP* polymorphism by quantifying the contemporary geographic distribution (i.e., phylogeography) of *PRNP* variants in hunter-harvested white-tailed deer (WTD; *Odocoileus virginianus*, N = 1433) distributed across Arkansas (USA), including a focal spot for CWD since detection of the disease in February 2016. Of these, *PRNP* variants associated with the well-characterized 96S non-synonymous substitution showed a significant increase in relative frequency among older CWD-positive cohorts. We interpreted this pattern as reflective of a longer life expectancy for 96S genotypes in a CWD-endemic region, suggesting either decreased probabilities of infection or reduced disease progression. Other variants showing statistical signatures of potential increased susceptibility, however, seemingly reflect an artefact of population structure. We also showed marked heterogeneity across the landscape in the prevalence of ‘reduced susceptibility’ genotypes. This may indicate, in turn, that differences in disease susceptibility among WTD in Arkansas are an innate, population-level characteristic that is detectable through phylogeographic analysis.

## Introduction

Chronic wasting disease (CWD) is a fatal neurodegenerative disorder that affects white-tailed deer (WTD; *Odocoileus virginianus*) and related cervids [1,2], with severe impacts on native wildlife, that also reverberate economically for recreational hunting and ancillary commercial enterprises [3,4]. Most CWD eradication efforts have proven unsuccessful thus far [5], leading to its continued spread and increased prevalence [6]. Given this, wildlife managers in many jurisdictions are responding by shifting long-term goals away from eradication and instead towards suppression, containment, and mitigation [7,8].

Several factors have impeded the eradication of CWD, including aspects of life history in both host and agent, as well as limited knowledge with regards to how these interact with environment to define CWD epidemiology. Pathogenicity and transmission, for example, occur via the structural transformation of a naturally occurring cellular prion protein (*PrP*^C^) into a misfolded ‘pathogenic’ isoform (*PrP*^SC^) [9]. The efficiency with which this occurs, coupled with an extensive incubation period [10], serve to confound proactive surveillance and management. Additionally, both vertical [11,12] and horizontal transmission [13,14] are seemingly involved, with prion persistence well established within ‘environmental reservoirs’ [15–17]. As a result, surveillance and monitoring are being increasingly used by state agencies to inform harvest and selective-removal-based management strategies to suppress the disease where it cannot be eradicated [18,19]. This is complicated particularly due to a potential for long-distance host dispersal [20–22], and prion ‘shedding’ by individuals with subclinical infections [23,24].

Environmental factors that act to reduce disease spread are of particular interest. For example, the capacity of soil as a reservoir for extra-corporeal prion persistence may hinge upon its composition [25]. Likewise, environmental factors that enhance the potential for WTD movements also may modulate CWD transmission among herds [26,27]. Considerable effort to date has focused on characterizing intrinsic susceptibility, especially with regard to the quantification of genetic polymorphisms that encode *PrP*^C^ (*PRNP*) [28–31]. One consistent approach is to identify those *PRNP* gene variants that are associated with reduced CWD susceptibility [32,33]. The amino acid composition of the resulting protein is thus assumed to impact disease progression [34], although the exact mechanism remains unclear.

Consistent among those variants reported to be associated with reduced CWD susceptibility is a non-synonymous mutation corresponding to an amino acid substitution at position 96 (i.e., from glycine to serine; hereafter 96G versus 96S). Inoculation studies employing this mutation have successfully delayed the progression of CWD in both WTD and proxies [13,35]. Our primary interest is to characterize if (and how) these *PRNP* alleles vary spatially [30,36], as one landscape-level axis that depicts a ‘resistance’ to CWD spread. We provide such an analysis by employing WTD sampled state-wide as a basis for the phylogeography of the *PRNP* gene (i.e., the geographic distribution of individuals associated with a gene genealogy [37]). We then examine spatial and age-structured patterns within this phylogeographic framework to ask several questions regarding the role of *PRNP* variants on population dynamics in CWD-endemic areas: Do ‘reduced susceptibility’ variants have an effect on survivorship (e.g., as one might expect if *PRNP* polymorphisms do indeed drive susceptibility)? If so, does this leave a *detectable* signature of biased fitness (e.g., as a result of increased survivability and thus potential reproductive output)? What are the impacts of *PRNP* polymorphism on population demographics? We address these questions at two spatial scales: 1) within a dense sampling of the CWD-focal area (Newton County, Arkansas) from which prevalence, and, presumably, measurable impacts on population demography are highest; and 2) state-wide, where sampling densities were lower, and in many areas within which CWD has not yet been detected (though we note that lack of detection does not mean lack of occurrence). The county-level spatial scale allowed us to examine demographic impacts within a recently detected outbreak in a novel area (Newton County), wherein dense sampling and high prevalence allow sufficient sampling for testing hypotheses of age structuring and selection on *PRNP* polymorphisms, while the state-level spatial scale allowed broad-scale analyses of heterogeneity and phylogeographic structuring. The combination of these two spatial scales allows for superimposing differential susceptibilities and fitness across a CWD-absent landscape which could in turn facilitate the creation of management scenarios to project and potentially mitigate disease spread.

## Results

### Data generation

**Figure 1.**
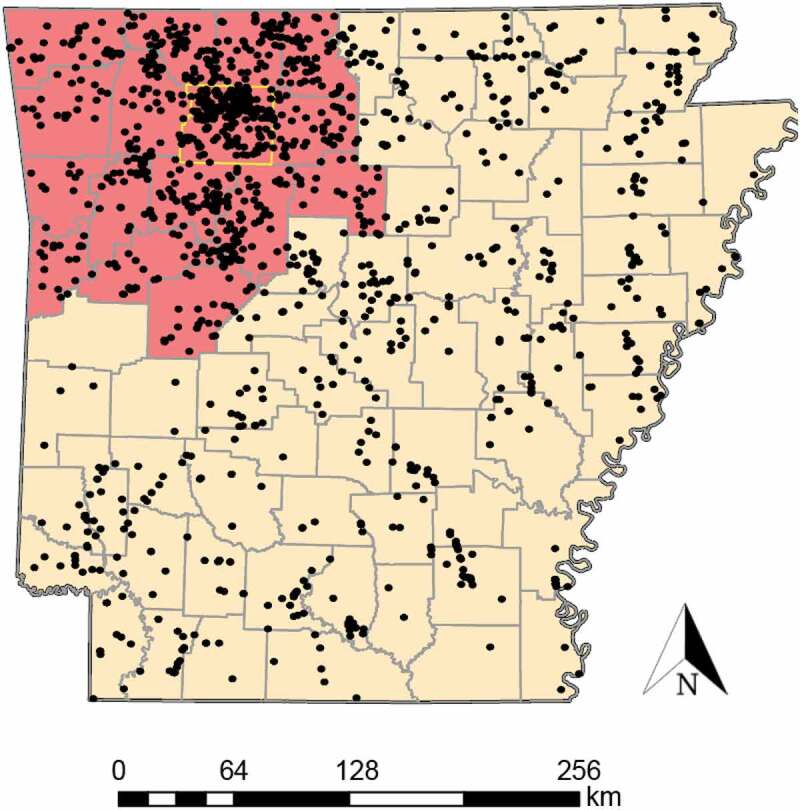
Sampling locations for white-tailed deer tissues from Arkansas evaluated in this study. The red shaded area indicates the 16 counties included in the 2019 Chronic Wasting Disease Management Zone (CWDMZ), with a yellow boundary surrounding a focal area encompassing Newton County. Black dots represent collection localities for each individual tissue. Note that the boundaries of the CWDMZ have since expanded

From 2016 to 2019, ear and tongue tissue samples were collected from 1,720 harvested WTD across 75 counties in Arkansas (Supplemental Table S1; Figure 1). Subsequently, tissue samples from 1,460 WTD were amplified and sequenced, yielding ~800 nucleotides of the *PRNP* gene and *PRNP^psg^* pseudogene. From these data, we obtained 1,433 sequences, of which 316 were obtained from Newton County, the CWD focal area. Sequences were trimmed to 720 unambiguously scored nucleotides, with 11 sites found to be polymorphic (Table 1). Three previously reported polymorphic sites (i.e., nt285, nt299 and nt372; Brandt et al., 2015, 2018) were found to be invariable in our data, whereas one additional site had a novel synonymous substitution (nt499, A/C). Three sites (i.e., nt286, nt367 and nt676) also reflected non-synonymous substitutions, corresponding to amino acid substitutions 96S, 122 T and 255 K, respectively.

**Table 1.** *PRNP* haplotypes tabulated from 1,433 white-tailed deer tissues collected from 75 counties in Arkansas (2016–2019). Haplotypes (Hap) are identified by letter (A-V) following Brandt et al. (2015, 2018). Haplotypes denoted as AR1-4 are previously unreported. Mutations that differ from haplotype A are shaded, with green indicating synonymous substitutions (no amino acid change) and blue as non-synonymous (amino acid changed in protein; NSS). Also listed are amino acid position and nucleotide site (Brandt et al. 2015, 2018). Polymorphisms not identified in prior studies are denoted as *

Hap	NSS	Amino Acid Position													
		20	51	81	95	**96**	99	108	**122**	124	126	146	166	185	**225**
		**Nucleotide Site**													
		60	153	243	285	**286**	299	324	**367**	372	378	438	499*	555	**676**
**A**		C	C	T	A	G	G	A	G	G	G	C	A	C	C
**B**		C	C	T	A	G	G	A	G	G	G	C	A	T	C
**C**	96S	C	C	T	A	**A**	G	A	G	G	G	C	A	T	C
**D**		C	T	T	A	G	G	A	G	G	G	C	A	C	C
**E**		C	C	T	A	G	G	A	G	G	G	T	A	C	C
**G**		T	C	T	A	G	G	A	G	G	G	C	A	C	C
**I**	96S	C	C	A	A	**A**	G	A	G	G	G	C	A	T	C
**J**		C	C	T	A	G	G	G	G	G	G	C	A	C	C
**K**	225 K	T	C	T	A	G	G	A	G	G	G	C	A	C	**A**
**L**	122 T	C	C	T	A	G	G	A	**A**	G	G	C	A	C	C
**O**		T	T	T	A	G	G	A	G	G	G	C	A	C	C
**P**	96S	C	C	T	A	**A**	G	A	G	G	G	C	A	C	C
**R**		C	T	T	A	G	G	A	G	G	G	C	A	T	C
**T**		C	T	T	A	G	G	A	G	G	A	C	A	C	C
**V**	96S	C	T	T	A	**A**	G	A	G	G	G	C	A	C	C
**AR1**		C	C	T	A	G	G	G	G	G	A	C	A	C	C
**AR2**	96S	C	C	T	A	**A**	G	A	G	G	G	C	C	T	C
**AR3**	96S	T	C	T	A	**A**	G	A	G	G	G	C	A	T	C
**AR4**		C	C	T	A	G	G	A	G	G	G	T	A	T	C

**Figure 2.**
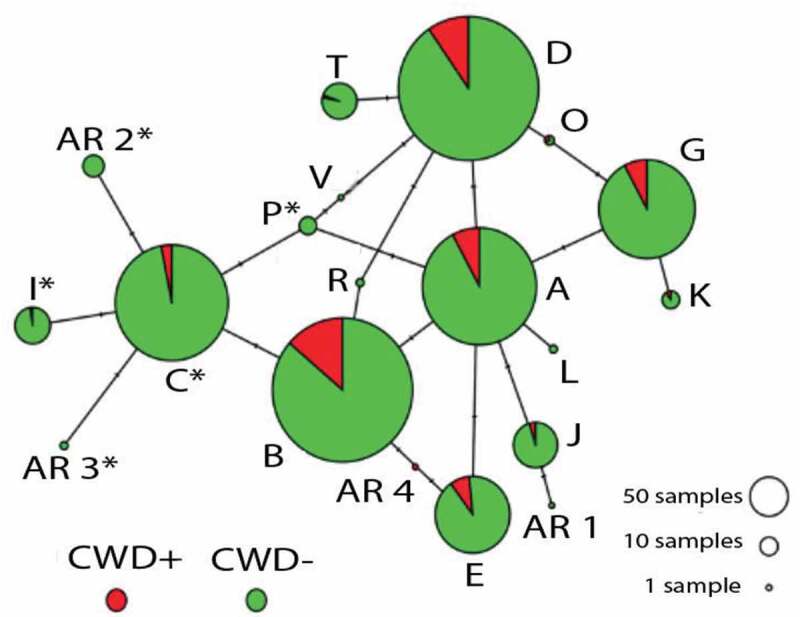
Haplotype network showing relationship of prion gene variants (*PRNP*) detected across 1,433 white-tailed deer collected from 75 counties in Arkansas (2016–2019). Data are based on sequence analysis of 720 nucleotides. Circles represent 20 haplotypes (=alleles) with size reflecting frequency of occurrence in entire data set (Table 2), and tick marks representing number of mutations (=nucleotide substitutions) distinguishing one from another (Table 1). Colour codes reflect relative frequency among CWD-positive (red) and CWD-negative/undetected (green). Letters correspond to haplotype names (per Brandt et al. 2015), with haplotypes unique to Arkansas indicated with numbers (AR#). Haplotypes sharing the 96S mutation are indicated with (*)

Haplotype phasing resulted in 20 distinct alleles (Table 1; Figure 2), 4 of which are novel, and 16 were previously documented in other states [29–31]. Two Arkansas haplotypes (AR 2 and AR 3) share the synonymous substitution 96S (nt286/A) associated with reduced CWD susceptibility (i.e., Haplotype C^30^). Three others (I, P and V) also share the 96S amino acid alteration (Table 1; Figure 2). However, all five were at low frequencies (<1%, except Haplotype I at 1.47%; Table 2), and thus were excluded from tests of CWD association.

**Table 2.** *PRNP* haplotype frequencies and odds-ratios, as associated with CWD status of white-tailed deer in Arkansas, 2016–2019. Haplotypes were derived from unphased sequences representing 720 nucleotides of the *PRNP* gene. Haplotypes (Hap) are identified by letter (A-V) following Brandt et al. (2015, 2018). Haplotypes denoted AR1-4 are previously unreported. Listed are total numbers (N) and relative frequency (%) and associated values for deer that were CWD-negative (-), CWD-positive (+) or untested (?). Odds Ratio (OR) reflects the relative representation of a haplotype in CWD-positive deer; OR>1 indicated over-representation, OR<1 under-representation. SE = standard error, CI = 95% confidence interval, Z = OR Z-score and *p*= OR *p*-value. Values in bold are significant

Hap	Counts					Frequency (%)								Odds Ratio					
	*N*	*N*(-)	*N*(+)		*N*(?)		%	%(-)		%(+)		%(?)			OR	SE	CI	Z	*p*
**A**	435	367		34	34	15.18			15.17		13.71		17.17	0.89		0.19	[0.61–1.30]	−0.61	0.54
**B**	657	536		90	31	22.92			22.15		36.29		15.66	**2.00**		**0.14**	**[1.52–2.64]**	**4.93**	**0.00**
**C**	426	376		13	37	14.86			15.54		5.24		18.69	**0.30**		**0.29**	**[0.17–0.53]**	**−4.14**	**0.00**
**D**	657	547		63	47	22.92			22.60		25.40		23.74	1.17		0.15	[0.86–1.58]	1.00	0.32
**E**	187	160		16	11	6.52			6.61		6.45		5.56	0.97		0.27	[0.57–1.66]	−0.10	0.92
**G**	309	266		24	19	10.78			10.99		9.68		9.60	0.87		0.22	[0.56–1.35]	−0.63	0.53
**I**	42	39		1	2	1.47			1.61		0.40		1.01	0.25		1.01	[0.03–1.81]	−1.38	0.17
**J**	65	59		3	3	2.27			2.44		1.21		1.52	0.49		0.60	[0.15–1.57]	−1.20	0.23
**K**	10	9		1	0	0.35			0.37		0.40		0.00	1.08		1.06	[0.14–8.60]	0.08	0.94
**L**	2	2		0	0	0.07			0.08		0.00		0.00	-		-	-	-	-
**O**	3	2		1	0	0.10			0.08		0.40		0.00	4.89		1.23	[0.44–54.2]	1.29	0.20
**P**	10	8		0	2	0.35			0.33		0.00		1.01	-		-	-	-	-
**R**	2	2		0	0	0.07			0.08		0.00		0.00	-		-	-	-	-
**T**	41	30		1	10	1.43			1.24		0.40		5.05	0.32		1.02	[0.04–2.38]	−1.11	0.27
**V**	1	1		0	0	0.03			0.04		0.00		0.00	-		-	-	-	-
**AR1**	1	0		0	1	0.03			0.00		0.00		0.51	-		-	-	-	-
**AR2**	15	14		0	1	0.52			0.58		0.00		0.51	-		-	-	-	-
**AR3**	2	2		0	0	0.07			0.08		0.00		0.00	-		-	-	-	-
**AR4**	1	0		1	0	0.03			0.00		0.40		0.00	-		-	-	-	-

Amplification and sequencing of the *PRNP^PSG^* pseudogene were successful in 30% of our samples (443 of 1,459). Our comparison of the 443 *PRNP* and *PRNP^PSG^* haplotypes indicated that nucleotide polymorphism at site nt413 did not represent a biological variant of the *PRNP* gene but was instead off-target amplification of the *PRNP^PSG^* pseudogene.

### PRNP haplotype frequencies

**Figure 3.**
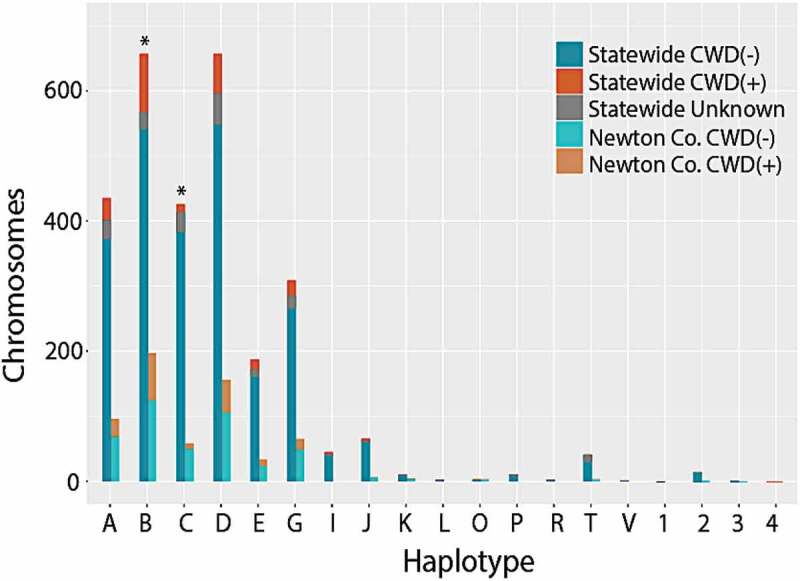
Stacked histogram of 2,866 *PRNP* haplotypes detected in white-tailed deer collected in Arkansas, 2016–2019. Haplotypes were determined by phasing individual genotypes derived from sequencing 1,433 deer across 720 bp of the *PRNP* gene. Letters (A through V) refer to haplotypes identified in Brandt et al. (2015), whereas numbers (1–4) are haplotypes unique to Arkansas, and thus previously unreported. Frequencies are plotted for all 1,433 samples (=statewide) as well as for a subset of 314 samples from Newton County (N = 628 chromosomes). Colour codes reflect frequency among CWD-positive (CWD+) and CWD-negative (CWD-) samples; unknown indicates samples that were not tested for CWD. For each haplotype, paired bars report values statewide (left) and for the Newton County subset (right)

Haplotype frequencies (Table 2 and S2) differed slightly from those reported in other states (e.g., Wisconsin and Illinois [30,31]). The four most frequent haplotypes in Illinois and Wisconsin (i.e., >10%), were also common among Arkansas samples (Figure 3; Haplotypes A-D). We also found that Haplotype A, most common in Illinois/Wisconsin at 30%, occurred in only 15% of our samples (Figure 3; Table 2). Haplotypes B and D were instead most common in our data (each at ~23%). Haplotype C, associated with reduced CWD susceptibility, was detected at a frequency of 15%, quite similar to that in Illinois/Wisconsin (17%). Two additional haplotypes (E and G) also occurred at high frequencies in Arkansas (7% and 11%, respectively), whereas they were found at <5% in the Illinois/Wisconsin [30]. Out of the 16 rare haplotypes with previously reported frequencies ≤1% (Haplotypes K-Z [30]), seven were also observed at low frequencies in Arkansas (i.e., Haplotypes K, L, O, P, R, T, and V). Haplotype frequencies also differed across counties (Supplemental Table S1), although we note that sampling effort was uneven across the state.

### Evidence for disease-mediated selection on PRNP variants

**Figure 4.**
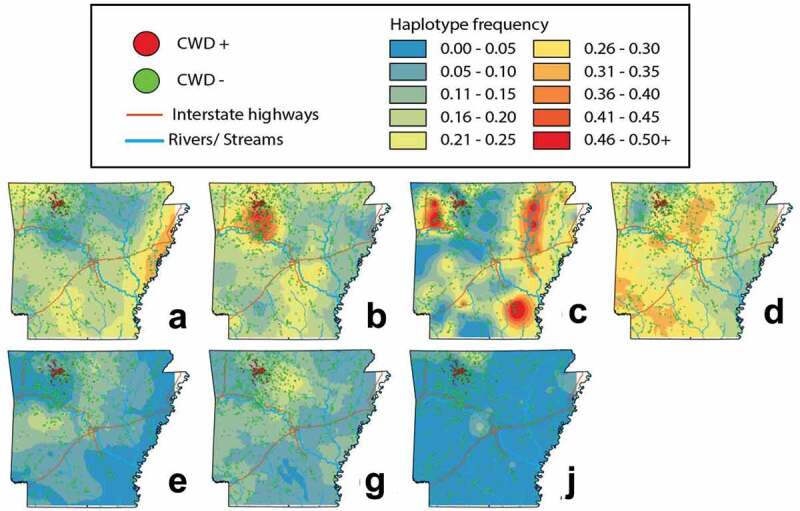
Topographies that represent interpolated haplotype frequencies for the *PRNP* gene in Arkansas. Frequency is depicted by colour, with blue reflecting low occurrence (0–5%) whereas red indicating 46–50+% of haplotypes were of this type

Haplotypes B and C did not occur in the same frequency within CWD-positive and CWD-negative deer (Table 2; Figure 3), and likewise were the most spatially heterogeneous of the observed haplotypes (Figure 4). Haplotype B was over-represented within CWD-positive deer, both state-wide (OR = 2.00, *p* = 0.000001), and in Newton County (OR = 1.43, *p*= 0.033). Haplotype C was under-represented within CWD-positive deer, both state-wide (OR = 0.30, *p* = 0.00003) and Newton County (OR = 0.42, *p* = 0.015).

**Figure 5.**
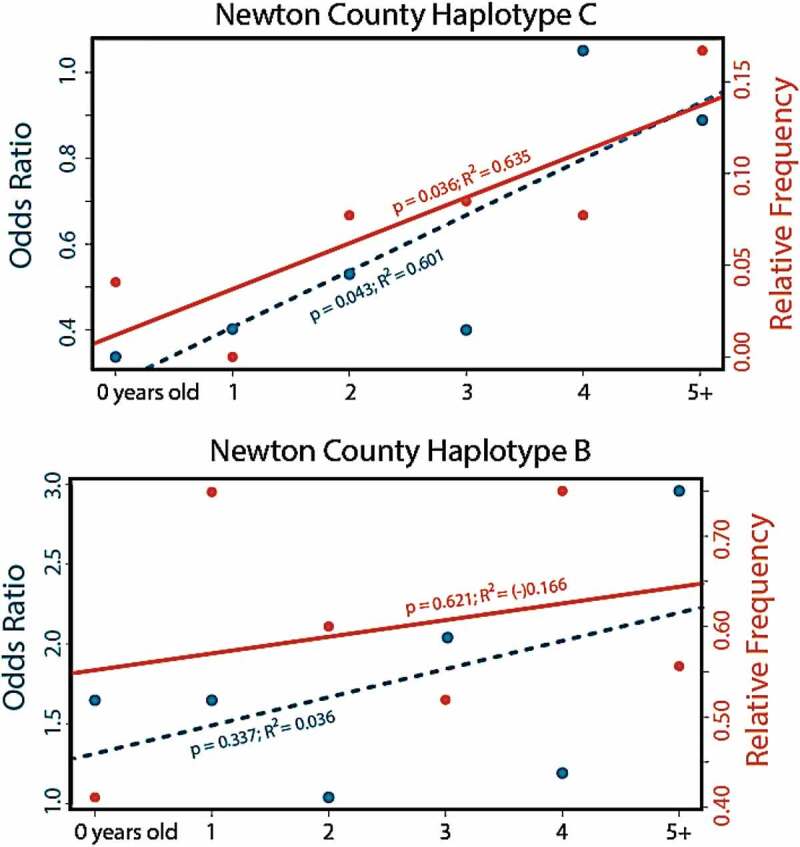
Relative frequency and odds ratio for two haplotypes of the prion gene *PRNP* haplotypes detected in white-tailed deer age cohorts (<1 year to 5+ years) sampled in Arkansas from 2016–2019. Prion gene variant Haplotype C (top panel) has been associated with reduced susceptibility to CWD, whereas Haplotype B (lower panel) has been associated with higher susceptibility (Brandt et al. 2018). Data are based on phased haplotypes derived from 720 nucleotides of the *PRNP* gene sequenced across 1,433 deer

If Haplotypes B and C influence CWD susceptibility, then their relative frequency of occurrence should vary among deer age-classes, reflecting a biased probability of reaching older age classes. We restricted our analyses to Newton County to ensure only deer from CWD-endemic locations were considered. We found the frequency of Haplotype C significantly more common in older deer, both when measured as relative allele frequency (*p*= 0.036; *R*^2^ = 0.635; Figure 5), and as an age-partitioned odds ratio (*p*= 0.043; *R*^2^ = 0.601; Figure 5), though we note that the sample size for older cohorts was lower than other cohorts (Figure S2). The increase in Haplotype C remained substantial even when we considered only the relative frequency of each haplotype among CWD-positive deer from Newton County (i.e., 4% of yearling/fawn haplotypes, and 17% of those from individuals older than 5; Supplemental Table **S**3). Among CWD-positive deer state-wide, Haplotype C was recorded at 5.24% of all sampled haplotypes (Table 2). By contrast, Haplotype B showed neither a discernible relationship regarding CWD status, nor relative frequencies across age classes (Figure 5; Supplemental Table **S**3).

## Discussion

Our diagnosis of variability in the *PRNP* gene across 1,433 WTD collected from 75 counties in Arkansas was comparable to that found in other states (Tables 1–2). Of the 20 haplotypes we detected, 16 were previously identified in other states, with those alleles at higher frequencies in Arkansas also being most common elsewhere [30]. Four novel variants were found at low frequencies (Table 2; Figure 2–3), with paralog artefacts as a source of this variation eliminated due to our sequencing of the pseudogene. Haplotype C, characterized by the 96S substitution, showed a significantly biased representation among CWD-positive deer, being under-represented in younger deer and over-represented in older CWD-positive deer (Figure 5). This suggests that 96S-individuals tend to live longer following CWD-infection than those with alternate genotypes. This could reflect either a reduced likelihood of contracting the disease or a slower disease progression following exposure.

We also found Haplotype B as significantly over-represented in CWD-positive deer (Table 2 and Figure 3) but failed to find a reciprocal impact on life expectancy (Figure 5). Instead, we found Haplotype B to be most extensive within the region where CWD is currently centred (Figure 4). This, in turn, may suggest heterogeneity in Haplotype B frequency represents an artefact of population structure, a well-known confounding variable recognized in trait-association studies [38–41]. We also note that decreased precision of ageing of white-tailed deer on the basis of tooth development and wear patterns may also contribute some variability, particularly with regard to haplotype frequencies among older cohorts [42–44]. We also cannot rule out the potential for linked variation as influencing the observed pattern in Haplotype B, however our results do not find any evidence for increased susceptibility of the variant. Using panels of nuclear markers, we suggest further study of patterns of spatial connectivity and population structure in wild WTD [45] as a means of separating potential spatial and phylogeographic drivers of haplotype frequencies from those driven by disease-mediated selection.

The recent detection of CWD in Arkansas may suggest an insufficient time to generate the genotype frequency differential we observed for Haplotype C (e.g., Figure 5). However, the prevalence rate in the Arkansas CWD Management Zone is suggestive that it was present in the landscape much earlier. Here, the putative peak prevalence rate of 22–30% (Middaugh and Riggs, pers. comm) points to a longer-standing epidemic. For example, Miller et. al [46] showed prevalence rates *in silico* to reach 1% in a 15–20 year span, and 15% in 37 to 50 years, using plausible transmission rates. This seemingly suggests that the time required to reach a prevalence rate of >22% is likely to be longer than 37–50 years. However, we note that true prevalence likely varies as a function of numerous demographic factors [47] and also likely exhibits a non-linear temporal trend [48], thus conclusions are highly dependent on the exact model parameterization [49].

### Management implications of PRNP variation

Structural variants of the *PrP* protein play a role in disease progression [28,34]. However, the exact mechanisms remain poorly understood. The primary variant we have implicated herein (i.e., 96S) as influencing disease susceptibility has been supported as such in laboratory settings. For example, Mathiason et al [23]. inoculated WTD with prion strains and examined time-to-detection (via saliva) across deer in multiple cohorts. Several infected individuals having the 96S prion gene variant remained undetected at 18 months post-inoculation, although this may represent an insufficient time for the necessary *in vivo* prion protein build-up. Race et al [35]. similarly inoculated transgenic mice expressing different white-tailed deer 96GG and 96SS *PRNP* genotypes and showed that this delay in disease progression also extended to heterozygotes (96GS genotype).

Despite compelling evidence for an inhibitory mechanism at the 96S allele, its use as a management tool remains unclear. Genetically guided selective breeding in domestic sheep (*Ovis aries*) has reduced scrapie incidence [50,51]. In a captive setting such an approach may be viable for WTD [35], although we note that the degree of protection offered by the 96S genotype to CWD is likely much lower than that seen among the most ‘protective’ genetic variants to scrapie in sheep [50]. However, captive deer maintained at high density and under genetic selection could also drive artificial selection for emergent prion strains with heightened pathogenicity in 96S deer, as well as potentially expanding the host range to include novel species [52].

The implication of 96S frequency with regard to the spread of CWD within and among herds is likewise uncertain. An important question is whether the potential for an increased incubation period [53] associated with 96S could also produce a similar extended period for asymptomatic and subclinical transmission/prion shedding [24,54]. If so, this could increase the probability of transmission by 96S deer, thus promoting its increase within populations (Supplemental Figure S3). An understanding of the prion growth kinetics across host genotypes is needed, as well as a more thorough understanding of prion strain evolution [55].

### Conclusion

Our results corroborate previous research conducted with WTD in Illinois and Wisconsin: reduced CWD susceptibility in *PRNP* variants associated with the non-synonymous 96S mutation [30,31]. We demonstrated that a common haplotype (Haplotype C) harbouring 96S increased in relative frequency when older CWD-positive cohorts were examined.

Management implications of this research requires further epidemiological understanding necessary to predict outcomes (Supplemental Figure S3). The next step in understanding the current distribution and future spread of CWD in Arkansas requires the characterization of context-specific factors, including 1) population structure as a potential driver of *PRNP* trends, 2) landscape features that modulate deer dispersal, population demography, and density, and 3) increased knowledge of epidemiology, including the interactions among context-specific factors [56]. We thus advocate for a landscape genomic framework for WTD as the next logical step to characterize CWD spread such that fine-scaled deer movement patterns can be more effectively parsed and interpreted [57].

## Materials and methods

### CWD surveillance and prevalence in Arkansa*s*

In October 2015, CWD was initially detected in Arkansas in a 2.5-year old female elk (*Cervus canadensis*) legally harvested near Pruitt in Newton County. In February 2016, a CWD-positive female WTD was found dead in Ponca in Newton County. During March 2016, biologists from the Arkansas Game and Fish Commission (AGFC) collected 266 WTD tissue samples within a 50,500 ha focal region. CWD prevalence was 23% and differed by gender (female = 20%, male = 32%). For these surveys, to include the samples collected for molecular work, ageing of deer was done by examination of tooth replacement and wear [58]. Although the method of ageing described by Severinghaus [58] is well established, we do note that it has reduced accuracy in older cohorts as compared with alternative methods using cementum annuli [44,59].

Subsequent state-wide monitoring, which included hunter-harvested and road-killed deer, identified CWD-positive individuals outside of the initial focal region. Additional state-wide sampling efforts, in conjunction with hunter harvested and bi-annual surveys, established a state-wide baseline for occurrence of CWD. As of April 2020, 818 WTD and 23 elk have tested positive for CWD. Given the incidence of confirmed CWD-positive deer, AGFC established a CWD Management Zone (CWDMZ) that included counties within a 16 km radius of identified positives. At the completion of this study (June 2019), the CWDMZ encompassed 19 counties of northwestern Arkansas (Figure 1).

There has been a concerted effort by the AGFC to proactively manage CWD prevalence and potential disease spread in Arkansas, which included hunting regulations that promote harvest of young male deer and increased harvest of female deer (e.g., removal of antler point restrictions and altered bag limits). Addition restrictions included prohibiting baiting and feeding to reduce grouping behaviour in deer and to hinder human-mediated transmission via hunting and subsequent removal of carcases from with the CWDMZ. The CWDMZ has subsequently been expanded to encompass the known distribution of CWD. During the 2018/2019 deer and elk hunting seasons, 246 additional CWD-positive cervids (241 WTD and five elk) were detected. Moreover, the AGFC has mandated a compulsory testing requirement for harvested elk, and a voluntary test for WTD, facilitated by a state-wide network of deer head drop-off locations.

### DNA extraction and amplification

Frozen ear or tongue tissue was homogenized with the TissueLyser II (QIAGEN© Corporation, Maryland, USA), with genomic DNA subsequently extracted using the QIAamp Fast DNA Tissue Kit protocol (QIAGEN© Corporation, Maryland, USA). To ascertain presence of high-quality genomic DNA (i.e., molecular weight >20kb), a 5 μl aliquot of the DNA extract was separated on a 2% agarose gel and visualized using GelGreen on a blue-light transilluminator (Gel Doc™ EZ Imager; Bio-Rad). DNA was then used as template material to amplify a coding section of the *PRNP* gene, following a protocol modified from previous studies [31,32]. For the functional *PRNP* gene, the forward primer (CWD-13) straddles Intron 2 and Exon 3, with the reverse primer (CWD-LA) located 850bp downstream [32]. To ascertain if the polymorphisms were indeed in the functional *PRNP* gene, we tested for the presence of the non-coding *PRNP* pseudogene (*PRNP^PSG^)* by using pseudogene primers 223 and 224 [33].

Amplifications for the functional *PRNP* gene and the *PRNP^PSG^* pseudogene were performed in 20 μl reactions consisting of 10 μl Qiagen HotStart Master Mix (1unit HotStartTaq DNA Polymerase, PCR Buffer with 3 mM MgCl_2_, and 400 µM of each dNTP), 8 μM each of the forward and reverse primer, 7.4 μl DNase-free water, and 1 μl of template DNA (~50-100 ng). Thermocyling protocols consisted of an initial denaturation step of 15 min at 95^o^C, followed by 10 cycles of 45 s denaturation at 95^o^C, 45 s annealing at 57^o^C, and 75 s extension at 72^o^C, 25 cycles of 30 s denaturation at 95^o^C, 30 s annealing at 55^o^C and 60 s extension at 72^o^C, completed with a final extension step of 5 min at 72^o^C.

If both *PRNP* and *PRNP^PSG^* amplified in a sample, then each was sequenced to identify the true polymorphism in the functional *PRNP* gene. Amplicons were enzymatically purified, sequenced using BigDye v. 3.1 (Applied Biosystem Inc., Forest City, CA) dye-terminator chemistry, and resolved on an ABI 3730XL GeneAnalyzer (University of Illinois Keck Centre for Functional and Comparative Genomics). Sequences were manually edited using Sequencher (v 5.4, Gene Codes, Ann Arbour MI) and aligned against a reference database of *PRNP* gene sequences obtained from the NCBI GenBank (Accession # AF156185.1; AY3600089.1; AY3600091.1).

### Haplotype phasing and network construction

Following alignment, sequences were phased to haplotypes (paired nuclear alleles) using the programPhase2 [60], which reconstructs haplotypes using a probabilistic model of linkage disequilibrium. Only haplotypes assigned with >90% posterior probability (N = 1,433) were retained. Scripts employed to format inputs and parse haplotype phasing are available at *github.com/tkchafin/haploTools*. Haplotypes were then categorized using a published nomenclature [31], with haplotype frequencies calculated globally, by county, and by CWD status. We constructed a haplotype network to visualize similarity amongst haplotype, using the median-joining algorithm employed by PopArt [61]. Scripts to formulate these input files are found at: *github.com/tkchafin/scripts*.

### Analysis of PRNP variants

We first applied spatial interpolation to examine the structure of *PRNP* haplotypes as distributed across the state. Haplotype frequencies were computed by first dividing the state into non-overlapping ‘pseudo-populations’ that contained between 5 and 10 sampling localities each. This was done because our state-wide sampling process lacked *a priori* information with regard to natural population structure. Our results yielded N = 211 polygons (Supplemental Figure 1). We then applied Empirical Bayesian Kriging in ArcMap v10.7.1 (Esri, Inc.) to interpolate our posterior probabilities.

To associate disease with *PRNP* variants, we followed prior studies [30,31] by computing odds ratios (OR). We first consider the probability of displaying an outcome (=CWD status) given the presence of a focal haplotype. An OR>1 = an over-representation of the focal haplotype among CWD-positive deer; an OR<1 indicates the focal haplotype is under-represented. We identified and evaluated haplotypes separately, by examining haplotype frequencies among age classes. We did so because a bias in relative representation can be driven by multiple factors such as population structure, which may drive a coincident relationship. Here, we assumed that if a haplotype indeed affects the probability of survival to adulthood in CWD-present regions, presumably by reducing disease risk and/or progression, then it should show a significant change in relative representation in older age groups.

## Supplementary Material

Supplemental Material

## References

[1] Williams ES, Young S. Spongiform encephalopathies in Cervidae. Rev Sci Tech. 1992;11:551–567. 1617203 10.20506/rst.11.2.611

[2] Mawdsley JR. Phylogenetic patterns suggest broad susceptibility to chronic wasting disease across Cervidae. Wildl Soc Bull. 2020;44:152–155.

[3] Bishop RC. The economic impacts of chronic wasting disease (CWD) in Wisconsin. Hum Dimens Wildl. 2004;9:181–192. .

[4] Arnot C, Laate E, Unterschultz J, et al. Chronic wasting disease (CWD) potential economic impact on cervid farming in Alberta. J Toxicol Environ Heal Part A Curr Issues. 2009;72:1014–1017. . 10.1080/15287390903084223 19697234

[5] Wasserberg G, Osnas EE, Rolley RE, et al. Host culling as an adaptive management tool for chronic wasting disease in white-tailed deer: A modelling study. J Appl Ecol. 2009;46:457–466. 19536340 10.1111/j.1365-2664.2008.01576.x PMC2695855

[6] Rivera NA, Brandt AL, Novakofski JE, et al. Chronic wasting disease in cervids: prevalence, impact and management strategies. Vet Med Res Reports. 2019;10:123–139. . 10.2147/VMRR.S197404 PMC6778748 31632898

[7] Uehlinger FD, Johnston AC, Bollinger TK, et al. Systematic review of management strategies to control chronic wasting disease in wild deer populations in North America. BMC Vet Res. 2016;12:173. 27549119 10.1186/s12917-016-0804-7 PMC4994292

[8] Miller MW, Fischer JR. The first five (or more) decades of chronic wasting disease: lessons for the five decades to come. Trans North Am Wildl Nat Resour Conf. 2016;81:1–12.

[9] Aguzzi A, Sigurdson C, Heikenwaelder M. Molecular mechanisms of prion pathogenesis. Annu Rev Pathol Mech Dis. 2008;3:11–40. 10.1146/annurev.pathmechdis.3.121806.154326 18233951

[10] Miller MW, Wild MA, Williams ES. Epidemiology of chronic wasting disease in captive Rocky Mountain elk. J Wildl Dis. 1998;34:532–538. 9706562 10.7589/0090-3558-34.3.532

[11] Selariu A, Powers JG, Nalls A, et al. In utero transmission and tissue distribution of chronic wasting disease-associated prions in free-ranging Rocky Mountain elk. J Gen Virol. 2015;96:3444–3455. 26358706 10.1099/jgv.0.000281 PMC4806583

[12] Nalls AV, McNulty E, Powers J, et al. Mother to offspring transmission of chronic wasting disease in Reeves’ Muntjac deer. PLoS One. 2013;8:e71844. 23977159 10.1371/journal.pone.0071844 PMC3743758

[13] Mathiason CK, Powers JG, Dahmes SJ, et al. Infectious prions in the saliva and blood of deer with chronic wasting disease. Science. 2006;314:133–136. 17023660 10.1126/science.1132661

[14] Potapov A, Merrill E, Pybus M, et al. Chronic wasting disease: possible transmission mechanisms in deer. Ecol Modell. 2013;250:244–257.

[15] Gough KC, Maddison BC. Prion transmission: prion excretion and occurrence in the environment. Prion. 2010;4:275–282. 20948292 10.4161/pri.4.4.13678 PMC3268960

[16] Johnson CJ, Phillips KE, Schramm PT, et al. Prions adhere to soil minerals and remain infectious. PLoS Pathog. 2006;2:e32. 16617377 10.1371/journal.ppat.0020032 PMC1435987

[17] Wiggins RC. Prion stability and infectivity in the environment. Neurochem Res. 2009;34:158–168. 18483857 10.1007/s11064-008-9741-6

[18] Potapov A, Merrill E, Pybus M, et al. Chronic wasting disease: transmission mechanisms and the possibility of harvest management. PLoS One. 2016;11:1–20. 10.1371/journal.pone.0151039 PMC4786122 26963921

[19] Manjerovic MB, Green ML, Mateus-Pinilla N, et al. The importance of localized culling in stabilizing chronic wasting disease prevalence in white-tailed deer populations. Prev Vet Med. 2014;113:139–145. Internet. . 24128754 10.1016/j.prevetmed.2013.09.011

[20] McCoy JE, Hewitt DG, Bryant FC. Dispersal by yearling male white-tailed deer and implications for management. J Wildl Manage. 2005;69:366–376.

[21] Long ES, Diefenbach DR, Rosenberry CS, et al. Multiple proximate and ultimate causes of natal dispersal in white-tailed deer. Behav Ecol. 2008;19:1235–1242.

[22] Kelly AC, Mateus-Pinilla NE, Douglas M, et al. Utilizing disease surveillance to examine gene flow and dispersal in white-tailed deer. J Appl Ecol. 2010;47:1189–1198.

[23] Mathiason CK, Hays SA, Powers J, et al. Infectious prions in pre-clinical deer and transmission of chronic wasting disease solely by environmental exposure. PLoS One. 2009;4:e5916. 10.1371/journal.pone.0005916 PMC2691594 19529769

[24] Tamguney G, Miller MW, Wolfe LL, et al. Asymptomatic deer excrete infectious prions in feces. Nature. 2009;461:529–532. 19741608 10.1038/nature08289 PMC3186440

[25] Saunders SE, Bartz JC, Bartelt-Hunt SL. Soil-mediated prion transmission: is local soil-type a key determinant of prion disease incidence? Chemosphere. 2012;87:661–667. 22265680 10.1016/j.chemosphere.2011.12.076

[26] Hemming-Schroeder E, Lo E, Salazar C, et al. Landscape genetics: A toolbox for studying vector-borne diseases. Front Ecol Evol. 2018;6:1–11.

[27] Kelly AC, Mateus-Pinilla NE, Brown W, et al. Genetic assessment of environmental features that influence deer dispersal: implications for prion-infected populations. Popul Ecol. 2014;56:327–340. .

[28] Robinson SJ, Samuel MD, O’Rourke KI, et al. The role of genetics in chronic wasting disease of North American cervids. Prion. 2012;6:153–162. 22460693 10.4161/pri.19640 PMC7082092

[29] Kelly AC, Mateus-Pinilla NE, Diffendorfer J, et al. Prion sequence polymorphisms and chronic wasting disease resistance in Illinois white-tailed deer (*Odocoileus virginianus*). Prion. 2008;2:28–36. 19164895 10.4161/pri.2.1.6321 PMC2634418

[30] Brandt AL, Green ML, Ishida Y, et al. Influence of the geographic distribution of prion protein gene sequence variation on patterns of chronic wasting disease spread in white-tailed deer (*Odocoileus virginianus*). Prion. 2018;12:204–215. 30041562 10.1080/19336896.2018.1474671 PMC6277178

[31] Brandt AL, Kelly AC, Green ML, et al. Prion protein gene sequence and chronic wasting disease susceptibility in white-tailed deer (*Odocoileus virginianus*). Prion. 2015;9:449–462. 26634768 10.1080/19336896.2015.1115179 PMC4964855

[32] Johnson C, Johnson J, Clayton M, et al. Prion protein gene heterogeneity in free-ranging white-tailed deer within the chronic wasting disease affected region of Wisconsin. J Wildl Dis. 2003;39:576–581. 14567218 10.7589/0090-3558-39.3.576

[33] O’Rourke KI, Spraker TR, Hamburg LK, et al. Polymorphisms in the prion precursor functional gene but not the pseudogene are associated with susceptibility to chronic wasting disease in white-tailed deer. J Gen Virol. 2004;85:1339–1346. 15105552 10.1099/vir.0.79785-0

[34] Johnson CJ, Herbst A, Duque-Velasquez C, et al. Prion protein polymorphisms affect chronic wasting disease progression. PLoS One. 2011;6(3):e17450. 21445256 10.1371/journal.pone.0017450 PMC3060816

[35] Race B, Meade-White K, Miller MW, et al. *In vivo* comparison of chronic wasting disease infectivity from deer with variation at prion protein residue 96. J Virol. 2011;85:9235–9238. 21697479 10.1128/JVI.00790-11 PMC3165848

[36] Miller WL, Walter WD. Spatial heterogeneity of prion gene polymorphisms in an area recently infected by chronic wasting disease. Prion. 2019;13:65–76. 30777498 10.1080/19336896.2019.1583042 PMC7000142

[37] Avise JC. Phylogeography: retrospect and prospect. J Biogeogr. 2009;36:3–15.

[38] Felsenstein J. Phylogenies and the comparative method. Am Nat. 1985;125:1–15.

[39] Hoggart CJ, Parra EJ, Shriver MD, et al. Control of confounding of genetic associations in stratified populations. Am J Hum Genet. 2003;72:1492–1504. 12817591 10.1086/375613 PMC1180309

[40] Pagel M. Detecting correlated evolution on phylogenies: A general method for the comparative analysis of discrete characters. Proc R Soc B Biol Sci. 1994;255:37–45.

[41] Carlson J, Kadie C, Mallal S, et al. Leveraging hierarchical population structure in discrete association studies. PLoS One. 2007;2(7):e591. 17611623 10.1371/journal.pone.0000591 PMC1899226

[42] Cook RL, Hart RV Ages assigned known-age Texas white-tailed deer: tooth wear versus cementum analysis. Proceedings of the Annual Conference of the Southeastern Association of Fish and Wildlife Agencies. 1979;33:195-201.

[43] Mitchell CJ, Smith WP. Reliability of techniques for determining age in southern white-tailed deer. J Tennessee Adacemy Sci. 1991;66:117–120.

[44] Gee KL, Holman JH, Causey MK, et al. Aging white-tailed deer by tooth replacement and wear: A critical evaluation of a time-honored technique. Wildl Soc Bull. 2002;30:387–393.

[45] Seabury CM, Oldeschulte DL, Bhattarai EK, et al. Accurate genomic predictions for chronic wasting disease in U.S. white-tailed deer. G3. 2020;10:1433–1441. 32122960 10.1534/g3.119.401002 PMC7144088

[46] Miller MW, Williams ES, McCarty CW, et al. Epizootiology of chronic wasting disease in free-ranging cervids in Colorado and Wyoming. J Wildl Dis. 2000;36:676–690. 11085429 10.7589/0090-3558-36.4.676

[47] Osnas EE, Heisey DM, Rolley RE, et al. Spatial and temporal patterns of chronic wasting disease: fine-scale mapping of a wildlife epidemic in Wisconsin. Ecol Appl. 2009;19:1311–1322. 19688937 10.1890/08-0578.1

[48] Miller MW, Conner MM. Epidemiology of chronic wasting disease in free-ranging mule deer: spatial, temporal, and demographic influences on observed prevalence patterns. J Wildl Dis. 2005;41:275–290. 16107661 10.7589/0090-3558-41.2.275

[49] Belsare AV, Gompper ME, Keller B, et al. An agent-based framework for improving wildlife disease surveillance: A case study of chronic wasting disease in Missouri white-tailed deer. Ecol Modell. 2020;417:108919. 32189826 10.1016/j.ecolmodel.2019.108919 PMC7079769

[50] Hagenaars TJ, Melchior MB, Bossers A, et al. Scrapie prevalence in sheep of susceptible genotype is declining in a population subject to breeding for resistance. BMC Vet Res. 2010;6:1–11. 20470415 10.1186/1746-6148-6-25 PMC2883980

[51] Goldmann W. PrP genetics in ruminant transmissible spongiform encephalopathies. Vet Res. 2008;39:30. 18284908 10.1051/vetres:2008010

[52] Herbst A, Velásquez CD, Triscott E, et al. Chronic wasting disease prion strain emergence and host range expansion. Emerg Infect Dis. 2017;23:1598–1600. 28820384 10.3201/eid2309.161474 PMC5572867

[53] Johnson C, Johnson J, Vanderloo JP, et al. Prion protein polymorphisms in white-tailed deer influence susceptibility to chronic wasting disease. J Gen Virol. 2006;87(7):2109–2114. 16760415 10.1099/vir.0.81615-0

[54] Stein RA. Super-spreaders in infectious diseases. Int J Infect Dis. 2011;15:e510–3. 21737332 10.1016/j.ijid.2010.06.020 PMC7110524

[55] Pöschel T, Brilliantov NV, Frömmel C. Kinetics of prion growth. Biophys J. 2003;85:3460–3474. 14645042 10.1016/S0006-3495(03)74767-5 PMC1303654

[56] White LA, Forester JD, Craft ME. Disease outbreak thresholds emerge from interactions between movement behavior, landscape structure, and epidemiology. Proc Natl Acad Sci U S A. 2018;115:7374–7379. 29941567 10.1073/pnas.1801383115 PMC6048514

[57] Blanchong JA, Robinson SJ, Samuel MD, et al. Application of genetics and genomics to wildlife epidemiology. J Wildl Manage. 2016;80:593–608.

[58] Severinghaus CW. Tooth development and wear as criteria of age in white-tailed deer. J Wildl Manage. 1949;13:195–216.

[59] Storm DJ, Samuel MD, Rolley RE, et al. Estimating ages of white-tailed deer: age and sex patterns of error using tooth wear-and-replacement and consistency of cementum annuli. Wildl Soc Bull. 2014;38:849–856.

[60] Stephens M, Smith NJ, Donnelly P. A new statistical method for haplotype reconstruction from population data. Am J Hum Genet. 2001;68:978–989. 11254454 10.1086/319501 PMC1275651

[61] Leigh JW, David B, Shinichi N. popart: full‐feature software for haplotype network construction. Methods Ecol Evol. 2015;6:1110–1116.

