# Supplementary material for: Age structuring and spatial heterogeneity in prion protein gene (PRNP) polymorphism in white-tailed deer
Source: Prion. 2020 Oct 20;14(1):238–48. doi: 10.1080/19336896.2020.1832947 (PMC7575228; doi:10.1080/19336896.2020.1832947)

**Supplemental Figures and Tables**

**Supplemental Table S1**: Samples per county and frequency of 20 *PRNP* haplotypes detected in 1,433 white-tailed deer collected in 75 counties in Arkansas from 2016-2019. Phased haplotypes were derived from sequence analysis of 720 nucleotides of the *PRNP* gene. Letters indicate haplotypes previously detected in other states, whereas numbers (1-4) indicate haplotypes unique to Arkansas. Variable sites of haplotypes are listed in Table 2. Samples that tested positive for CWD (+) are listed separately for those counties where CWD was detected.

| **County** | **N** | **Haplotype** | | | | | | | | | | | | | | | | | | |
| --- | --- | --- | --- | --- | --- | --- | --- | --- | --- | --- | --- | --- | --- | --- | --- | --- | --- | --- | --- | --- |
|  |  | **A** | **B** | **C** | **D** | **E** | **G** | **I** | **J** | **K** | **L** | **O** | **P** | **R** | **T** | **V** | **1** | **2** | **3** | **4** |
| **Arkansas** | **11** | 4 | 5 | 5 | 4 | - | 3 | - | - | - | - | - | - | - | 1 | - | - | - | - | - |
| **Ashley** | **1** | 1 | - | - | - | - | - | - | 1 | - | - | - | - | - | - | - | - | - | - | - |
| **Baxter** | **16** | 3 | 7 | 3 | 8 | 4 | 6 | - | 1 | - | - | - | - | - | - | - | - | - | - | - |
| **Benton** | **40** | 25 | 19 | 3 | 16 | 5 | 5 | 1 | 4 | - | - | - | 1 | - | 1 | - | - | - | - | - |
| **Boone** | **33** | 6 | 24 | 8 | 7 | 5 | 8 | - | 7 | - | 1 | - | - | - | - | - | - | - | - | - |
| ***Boone(+)*** | ***2*** | - | - | - | 3 | - | 1 | - | - | - | - | - | - | - | - | - | - | - | - | - |
| **Bradley** | **2** | 1 | - | 2 | - | - | - | - | - | - | - | - | 1 | - | - | - | - | - | - | - |
| **Carroll** | **33** | 18 | 13 | 4 | 11 | 4 | 7 | - | 6 | - | - | - | - | - | 3 | - | - | - | - | - |
| ***Carroll(+)*** | ***16*** | 5 | 12 | 2 | 5 | 3 | 5 | - | - | - | - | - | - | - | - | - | - | - | - | - |
| **Calhoun** | **14** | 4 | 9 | 1 | 6 | 3 | 2 | - | 1 | - | - | - | 1 | - | 1 | - | - | - | - | - |
| **Cleburne** | **8** | 2 | 2 | 2 | 4 | 2 | 4 | - | - | - | - | - | - | - | - | - | - | - | - | - |
| **Chicot** | **4** | 3 | - | 1 | 2 | - | 1 | 1 | - | - | - | - | - | - | - | - | - | - | - | - |
| **Clark** | **13** | 2 | 5 | 6 | 6 | 1 | 1 | 3 | 1 | - | - | - | 1 | - | - | - | - | - | - | - |
| **Conway** | **10** | 1 | 4 | 1 | 6 | 3 | 5 | - | - | - | - | - | - | - | - | - | - | - | - | - |
| **Columbia** | **4** | 1 | 1 | - | 4 | - | 2 | - | - | - | - | - | - | - | - | - | - | - | - | - |
| **Craighead** | **12** | 5 | 1 | 5 | 7 | 1 | 3 | - | 1 | - | - | - | - | - | 1 | - | - | - | - | - |
| **Cross** | **9** | 4 | 4 | 3 | 5 | - | 1 | 1 | - | - | - | - | - | - | - | - | - | - | - | - |
| **Crittenden** | **8** | 5 | 1 | 5 | 4 | - | 1 | - | - | - | - | - | - | - | - | - | - | - | - | - |
| **Crawford** | **9** | 1 | 3 | 5 | 6 | - | 1 | - | - | 2 | - | - | - | - | - | - | - | - | - | - |
| **Clay** | **14** | 7 | 4 | 7 | 3 | 1 | 2 | 1 | - | - | - | - | 1 | - | 2 | - | - | - | - | - |
| **Dallas** | **9** | 6 | 3 | 1 | 3 | 3 | 1 | 1 | - | - | - | - | - | - | - | - | - | - | - | - |
| **Drew** | **14** | 4 | 2 | 16 | 3 | 1 | 2 | - | - | - | - | - | - | - | - | - | - | - | - | - |
| **Desha** | **12** | 8 | 4 | 5 | 4 | - | 1 | - | - | - | - | - | - | - | 1 | - | 1 | - | - | - |
| **Faulkner** | **14** | 5 | 1 | 2 | 11 | 4 | 1 | - | 1 | - | - | - | 1 | 2 | - | - | - | - | - | - |
| **Franklin** | **33** | 6 | 23 | 29 | 1 | - | 5 | - | - | - | - | - | - | - | - | - | - | 2 | - | - |
| **Fulton** | **21** | 3 | 8 | 4 | 12 | 4 | 5 | - | 1 | - | - | - | - | - | 5 | - | - | - | - | - |
| **Garland** | **16** | 6 | 5 | 6 | 5 | 4 | 1 | 3 | - | - | - | - | - | - | - | - | - | 2 | - | - |
| **Greene** | **12** | 1 | 8 | 7 | - | - | 2 | 2 | 1 | - | - | - | - | - | 3 | - | - | - | - | - |
| **Grant** | **10** | 4 | 3 | 3 | 7 | - | 2 | - | - | - | - | - | - | - | 1 | - | - | - | - | - |
| **Hampstead** | **10** | 6 | 3 | - | 5 | 2 | 1 | 3 | - | - | - | - | - | - | - | - | - | - | - | - |
| **Howard** | **13** | - | 3 | 6 | 11 | 2 | 2 | 1 | - | - | - | - | - | - | 1 | - | - | - | - | - |
| **Hot Springs** | **5** | 2 | 2 | 2 | 1 | 1 | 2 | - | - | - | - | - | - | - | - | - | - | - | - | - |
| **Independence** | **10** | - | 3 | 5 | 4 | 1 | 4 | - | - | - | - | - | - | - | 1 | - | - | 2 | - | - |
| **Izard** | **9** | - | 2 | - | 7 | 6 | 2 | - | - | - | - | - | - | - | - | - | - | 1 | - | - |
| **Jackson** | **2** | - | - | 3 | 1 | - | - | - | - | - | - | - | - | - | - | - | - | - | - | - |
| **Jefferson** | **13** | 6 | 7 | 2 | 5 | 1 | 4 | - | 1 | - | - | - | - | - | - | - | - | - | - | - |
| **Johnson** | **45** | 10 | 33 | 19 | 20 | 1 | 4 | 1 | - | - | - | - | 1 | - | - | - | - | 1 | - | - |
| **Lafayette** | **15** | 5 | 3 | 7 | 5 | 1 | 4 | 1 | - | - | - | - | - | - | 3 | - | - | 1 | - | - |
| **Lincoln** | **9** | 3 | 3 | 5 | 4 | - | 1 | - | 2 | - | - | - | - | - | - | - | - | - | - | - |
| **Lee** | **8** | 4 | 6 | 3 | 1 | - | 1 | - | - | 1 | - | - | - | - | - | - | - | - | - | - |
| **Lonoke** | **13** | 6 | 4 | 5 | 7 | 3 | 1 | - | - | - | - | - | - | - | - | - | - | - | - | - |
| **Logan** | **41** | 12 | 15 | 3 | 26 | 12 | 13 | - | 1 | - | - | - | - | - | - | - | - | - | - | - |
| **Little River** | **10** | 1 | 4 | 5 | 8 | 1 | 1 | - | - | - | - | - | - | - | - | - | - | - | - | - |
| **Lawrence** | **16** | 1 | 3 | 16 | 2 | 4 | 3 | - | - | - | - | - | - | - | 3 | - | - | - | - | - |
| **Madison** | **36** | 9 | 18 | 24 | 6 | 3 | 6 | 1 | 2 | - | - | - | - | - | 2 | - | - | 1 | - | - |
| ***Madison(+)*** | ***4*** | 1 | 1 | 1 | - | 2 | - | 1 | 2 | - | - | - | - | - | - | - | - | - | - | - |
| **Marion** | **40** | 10 | 12 | 11 | 12 | 10 | 7 | - | 14 | 1 | - | - | 2 | - | 1 | - | - | - | - | - |
| **Miller** | **8** | 3 | 5 | 2 | 1 | 1 | 2 | - | - | - | - | - | - | - | 2 | - | - | - | - | - |
| **Monroe** | **9** | 4 | 1 | 5 | 3 | 4 | 1 | - | - | - | - | - | - | - | - | - | - | - | - | - |
| **Montgomery** | **2** | 1 | 1 | 2 | - | - | - | - | - | - | - | - | - | - | - | - | - | - | - | - |
| **Mississippi** | **2** | 2 | - | 1 | - | - | - | - | 1 | - | - | - | - | - | - | - | - | - | - | - |
| **Nevada** | **14** | 7 | - | 6 | 10 | 2 | 3 | - | - | - | - | - | - | - | - | - | - | - | - | - |
| **Newton** | **216** | 68 | 124 | 49 | 105 | 23 | 48 | - | 5 | 3 | - | 2 | - | - | 2 | - | - | 2 | 1 | - |
| ***Newton(+)*** | ***100*** | 28 | 75 | 10 | 54 | 11 | 17 | - | 1 | 1 | - | 1 | - | - | 1 | - | - | - | - | 1 |
| **Ouachita** | **14** | 4 | 3 | 3 | 13 | 3 | 1 | 1 | - | - | - | - | - | - | - | - | - | - | - | - |
| **Perry** | **8** | 4 | 3 | 2 | 2 | - | 2 | 2 | 1 | - | - | - | - | - | - | - | - | - | - | - |
| **Phillips** | **9** | 8 | 1 | 1 | 1 | - | 5 | - | 1 | - | - | - | - | - | - | - | - | - | 1 | - |
| **Pike** | **8** | 2 | 3 | 2 | 5 | - | 2 | 2 | - | - | - | - | - | - | - | - | - | - | - | - |
| **Polk** | **3** | 2 | - | - | - | 2 | 1 | 1 | - | - | - | - | - | - | - | - | - | - | - | - |
| **Poinsett** | **13** | 7 | 1 | 5 | 6 | 2 | 3 | - | 1 | - | - | - | - | - | - | - | - | 1 | - | - |
| **Pope** | **61** | 3 | 48 | 9 | 41 | 3 | 15 | 3 | - | - | - | - | - | - | - | - | - | - | - | - |
| ***Pope(+)*** | ***1*** | - | 1 | - | - | - | 1 | - | - | - | - | - | - | - | - | - | - | - | - | - |
| **Prairie** | **11** | 3 | 5 | 7 | 3 | - | 1 | 1 | - | 2 | - | - | - | - | - | - | - | - | - | - |
| **Pulaski** | **11** | 3 | 3 | 4 | 5 | 3 | 3 | 1 | - | - | - | - | - | - | - | - | - | - | - | - |
| **Randolph** | **8** | 3 | 3 | 5 | 3 | - | - | - | 1 | - | - | - | - | - | 1 | - | - | - | - | - |
| **Saline** | **12** | 4 | 4 | 4 | 1 | 3 | 3 | 1 | 2 | - | - | - | - | - | 2 | - | - | - | - | - |
| **Sebastian** | **16** | 6 | 3 | 2 | 12 | 3 | 5 | 1 | - | - | - | - | - | - | - | - | - | - | - | - |
| ***Sebastian(+)*** | ***1*** | - | 1 | - | 1 | - | - | - | - | - | - | - | - | - | - | - | - | - | - | - |
| **Scott** | **2** | 1 | 2 | - | 1 | - | - | - | - | - | - | - | - | - | - | - | - | - | - | - |
| **Searcy** | **42** | 8 | 15 | 7 | 31 | 5 | 16 | - | 1 | - | - | - | 1 | - | - | - | - | - | - | - |
| **St Francis** | **8** | 4 | 4 | 3 | 5 | - | - | - | - | - | - | - | - | - | - | - | - | - | - | - |
| **Sharp** | **15** | 3 | 6 | 7 | 8 | 1 | 1 | - | 1 | - | - | - | - | - | 3 | - | - | - | - | - |
| **Stone** | **15** | 6 | 5 | 3 | 6 | 1 | 9 | - | - | - | - | - | - | - | - | - | - | - | - | - |
| **Sevier** | **10** | 5 | 1 | 2 | 5 | 1 | 4 | 2 | - | - | - | - | - | - | - | - | - | - | - | - |
| **Union** | **7** | 3 | 3 | 1 | 4 | 1 | 1 | - | - | - | - | - | - | - | - | - | - | 1 | - | - |
| **VanBuren** | **33** | 7 | 10 | 2 | 21 | 8 | 14 | 2 | - | - | 1 | - | - | - | - | 1 | - | - | - | - |
| **Washington** | **18** | 7 | 5 | 11 | 9 | - | 2 | 1 | 1 | - | - | - | - | - | - | - | - | - | - | - |
| **Woodruff** | **9** | 1 | 2 | 7 | 2 | 1 | 4 | - | - | - | - | - | - | - | - | - | - | 1 | - | - |
| **White** | **8** | 2 | 4 | 6 | - | 1 | 2 | - | 1 | - | - | - | - | - | - | - | - | - | - | - |
| **Yell** | **40** | 9 | 22 | 5 | 21 | 10 | 9 | 3 | 1 | - | - | - | - | - | - | - | - | - | - | - |
| **TOTAL** | **1433** | **435** | **657** | **426** | **657** | **187** | **309** | **42** | **65** | **10** | **2** | **3** | **10** | **2** | **41** | **1** | **1** | **15** | **2** | **1** |

**Supplemental Table S2:** Association of *PRNP* haplotype frequencies and odds ratio with CWD status for Newton County, Arkansas. Haplotypes were derived from unphased sequences of 720 nucleotides of the *PRNP* gene. Haplotype as indicated by letters were also reported by Brandt et al. (2015, 2018), whereas AR_# indicates a haplotype unique to Arkansas. Listed are total numbers (N), relative frequency f(%), and values for deer that tested either CWD-negative (-), CWD-positive (+) or were untested (?). Odds Ratio (OR) reflects relative representation of a haplotype in CWD+ deer, with OR>1 = over-representation and OR<1 = under-representation; SE= standard error, CI= 95% confidence interval, Z(OR)= Z-score and p(OR)= probability. **Values in bold are significant**.

| **Hap** | **Counts** | | | **Frequency (%)** | | | | | **Odds Ratio** | | | | |
| --- | --- | --- | --- | --- | --- | --- | --- | --- | --- | --- | --- | --- | --- |
|  | N | N(-) | N(+) | f% | f%(-) | | f%(+) | | OR | SE | CI | Z(OR) | p(OR) |
| **A** | 95 | 68 | 27 | 15.1 | | 15.7 | | 13.8 | 0.86 | 0.25 | [053-1.38] | -0.64 | 0.52 |
| **B** | 197 | 124 | 73 | 31.4 | | 28.7 | | 37.2 | **1.47** | **0.18** | **[1.03-2.11]** | **2.13** | **0.03** |
| **C** | 59 | 49 | 10 | 9.39 | | 11.3 | | 5.1 | **0.42** | **0.36** | **[0.21-0.85]** | **-2.42** | **0.02** |
| **D** | 158 | 105 | 53 | 25.2 | | 24.3 | | 27 | 1.15 | 0.20 | [0.79-1.70] | 0.73 | 0.46 |
| **E** | 34 | 23 | 11 | 5.41 | | 5.32 | | 5.61 | 1.06 | 0.38 | [0.50-2.21] | 0.15 | 0.88 |
| **G** | 65 | 48 | 17 | 10.4 | | 11.1 | | 8.67 | 0.76 | 0.30 | [0.43-1.35] | -0.93 | 0.35 |
| **I** | - | - | - | - | | - | | - | - | - | - | - | - |
| **J** | 6 | 5 | 1 | 0.96 | | 1.16 | | 0.51 | 0.44 | 1.10 | [0.05-3.77] | -0.75 | 0.45 |
| **K** | 4 | 3 | 1 | 0.64 | | 0.69 | | 0.51 | 0.73 | 1.16 | [0.08-7.09] | -0.27 | 0.79 |
| **L** | - | - | - | - | | - | | - | - | - | - | - | - |
| **O** | 3 | 2 | 1 | 0.48 | | 0.46 | | 0.51 | 1.10 | 1.23 | [0.10-12.23] | 0.08 | 0.94 |
| **P** | - | - | - | - | | - | | - | - | - | - | - | - |
| **R** | - | - | - | - | | - | | - | - | - | - | - | - |
| **T** | 3 | 2 | 1 | 0.48 | | 0.46 | | 0.51 | 1.10 | 1.23 | [0.10-12.23] | 0.08 | 0.94 |
| **V** | - | - | - | - | | - | | - | - | - | - | - | - |
| **AR_1** | - | - | - | - | | - | | - | - | - | - | - | - |
| **AR_2** | 2 | 2 | - | 0.32 | | 0.46 | | - | - | - | - | - | - |
| **AR_3** | 1 | 1 | - | 0.16 | | 0.23 | | - | - | - | - | - | - |
| **AR_4** | 1 | - | 1 | 0.16 | | - | | 0.51 | - | - | - | - | - |
| Total | 628 | 432 | 196 |  | |  | |  |  |  |  |  |  |

**Supplemental Table S3:** *PRNP* haplotype frequencies and odds ratio for six age classes of white-tailed deer collected in Newton County, Arkansas from 2016-2019. Relative frequencies and odds ratio are shown for (A) Haplotype C (associated with reduced CWD susceptibility), and (B) Haplotype B (associated with increased CWD susceptibility). Relative frequency of CWD-negative [f(-)] and CWD-positive [f(+)] samples were calculated by dividing numbers CWD(+/-) with C or B, respectively, then by negatives w/ other haplotypes.

**(A) Haplotype C**

| **Age** | **N** | **N(-)** | **N(+)** | **f(-)** | **f(+)** | **OR** |
| --- | --- | --- | --- | --- | --- | --- |
| **FAWN** | 9 | 8 | 1 | 0.13 | 0.04 | 0.34 |
| **Y1** | 5 | 5 | 0 | 0.08 | 0.00 | 0.40 |
| **Y2** | 22 | 18 | 4 | 0.15 | 0.08 | 0.53 |
| **Y3** | 16 | 13 | 3 | 0.21 | 0.09 | 0.40 |
| **Y4** | 4 | 3 | 1 | 0.07 | 0.08 | 1.05 |
| **Y5** | 8 | 6 | 2 | 0.19 | 0.17 | 0.89 |
| **Total** | 64 | 53 | 11 |  |  |  |

**(B) Haplotype B**

| **Age** | **N** | **N(-)** | **N(+)** | **f(-)** | **f(+)** | **OR** |
| --- | --- | --- | --- | --- | --- | --- |
| **FAWN** | 21 | 14 | 7 | 0.25 | 0.41 | 1.65 |
| **Y1** | 26 | 20 | 6 | 0.45 | 0.75 | 1.65 |
| **Y2** | 73 | 52 | 21 | 0.58 | 0.60 | 1.04 |
| **Y3** | 28 | 15 | 13 | 0.25 | 0.52 | 2.05 |
| **Y4** | 23 | 17 | 6 | 0.63 | 0.75 | 1.19 |
| **Y5** | 11 | 6 | 5 | 0.19 | 0.56 | 2.96 |
| **Total:** | 182 | 124 | 58 |  |  |  |

**Supplemental Figure S1:** Spatial distribution of 211 non-overlapping polygons in the state of Arkansas (U.S.A.). Each included 5-10 sampling locations. Closed red and green circles represent 1,433 white-tailed deer samples employed to compute prion gene variant (*PRNP* haplotype) frequencies for interpolation. Red circles = CWD+; green circles = CWD-


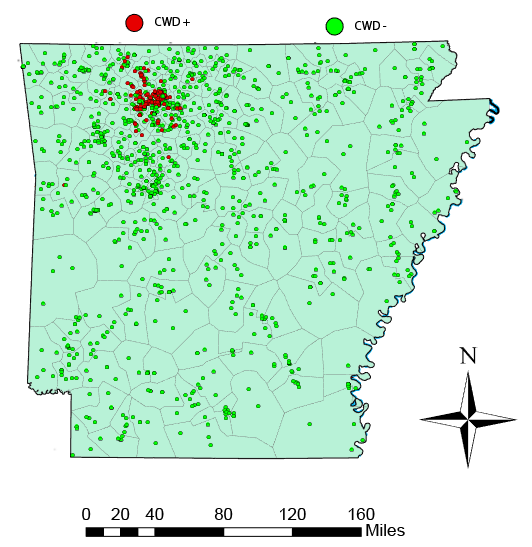


**Supplemental Figure S2:** Age and sex distribution for *N*=1,433 white-tailed deer tissue samples for which *PRNP* haplotype data was generated.

**
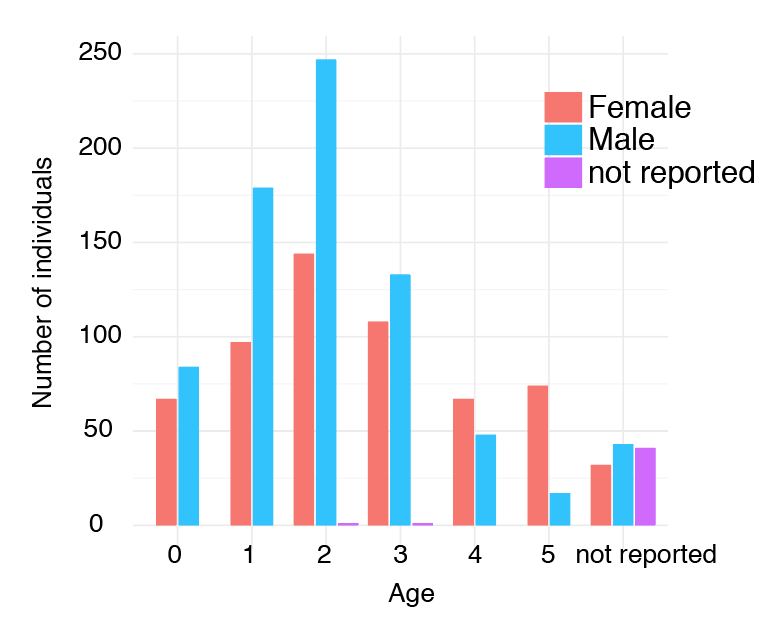
**

**Supplementary Figure S3:** Results of a conceptual model depicting prion conversion rates for *PrP*^C^ variants 96G and 96S. The diagram demonstrates prion conversion of 96G and 96S (reduced susceptibility) prion gene (=*PRNP*) variants. Theoretical prion protein (=*PrP*) mass thresholds were imposed: Ta = time of symptomatic expression; and Tb = theoretical time after which individuals remain asymptomatic. Individual-individual transmission is possible through direct contact (saliva) or shedding (e.g. feces). The time interval for *asymptomatic spread* (e.g., prion shedding from a subclinical individual) extends prior to *PrP* conversion surpassing threshold Tb, but not extending beyond Ta.


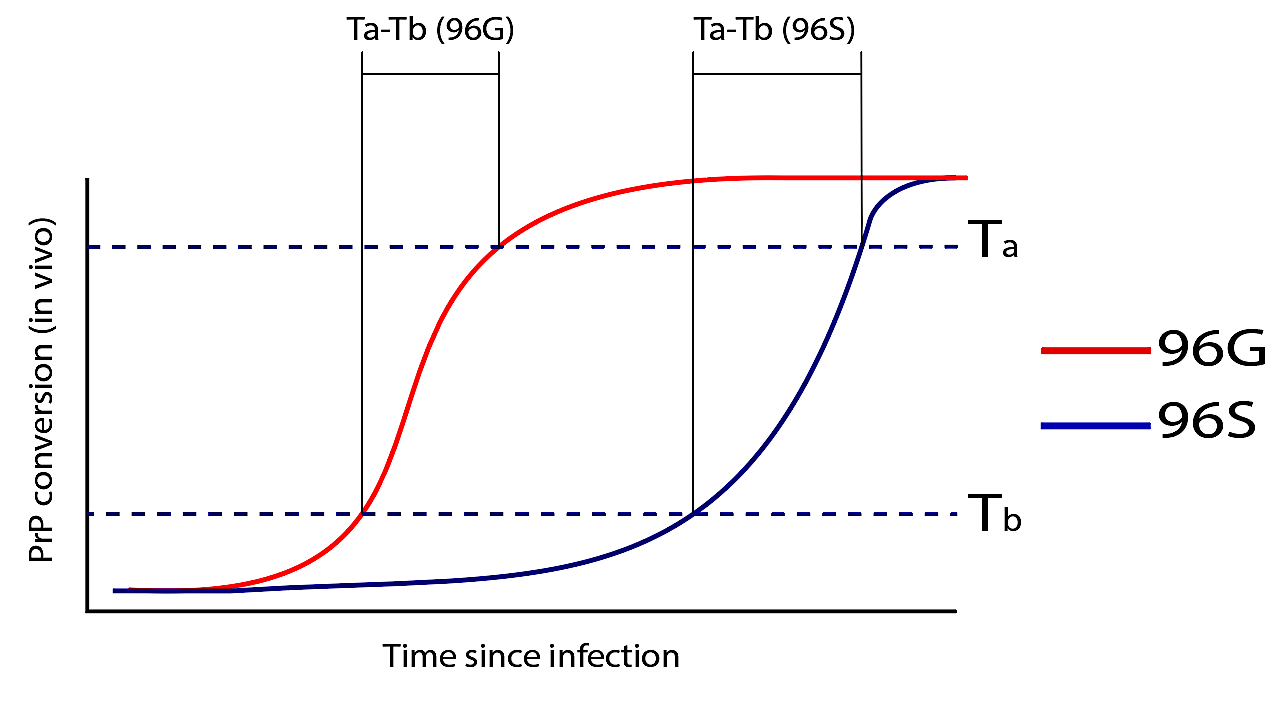

Supplement: Supplemental Material [file KPRN_A_1832947_SM9401.docx]
